# Diagnosis of Pelvic Actinomycosis and Fusobacterium Infection Using 16S rRNA Gene Analysis for Formalin-Fixed Paraffin-Embedded (FFPE) Tissue After Intrauterine Device (IUD) Removal

**DOI:** 10.7759/cureus.91208

**Published:** 2025-08-28

**Authors:** Erika Kitakura, Toshimichi Onuma, Makoto Orisaka, Yoshio Yoshida

**Affiliations:** 1 Obstetrics and Gynecology, Fukui Prefectural Hospital, Fukui, JPN; 2 Obstetrics and Gynecology, University of Fukui, Fukui, JPN

**Keywords:** 16s rrna, actinomyces israelii, fusobacterium nucleatum, gynecological malignancy, intrauterine device, mixed anaerobic infection, next-generation sequencing, pelvic infection

## Abstract

Severe pelvic infections present a diagnostic challenge owing to the clinical and radiological resemblance to malignancy. Pelvic actinomycosis has been implicated in the exacerbation of other anaerobic infections. Herein, we present the case of a 65-year-old woman who developed severe pelvic actinomycosis and *Fusobacterium *infection eight months after intrauterine device (IUD) removal.

The patient presented with lower abdominal pain. Imaging revealed an enlarged uterus with extensive parametrial invasion, bilateral hydronephrosis, and rectal stenosis, mimicking uterine sarcoma. Laboratory tests revealed elevation in the levels of inflammatory markers and renal dysfunction. Surgery was performed under suspicion of malignancy; however, histopathological examination revealed no malignant cells. Grocott staining identified filamentous organisms consistent with *Actinomyces*; however, conventional cultures of ascites were negative. Next-generation sequencing (NGS) of 16S ribosomal RNA (rRNA) genes extracted from the formalin-fixed paraffin-embedded (FFPE) tissue revealed a mixed infection of *Actinomyces israelii* and *Fusobacterium nucleatum*. The hydronephrosis resolved after treatment, with no recurrence by the six-month follow-up.

This case highlights the fact that pelvic actinomycosis and *Fusobacterium *infection can develop even after IUD removal, with cases potentially mimicking gynecological malignancy. To our knowledge, this is the first reported case of pelvic actinomycosis and *Fusobacterium *infection diagnosed by 16S rRNA gene analysis for FFPE tissue using NGS in a patient after IUD removal. Mixed infection involving *Actinomyces israelii* and *Fusobacterium nucleatum* may increase pathogenicity through synergistic interactions. When conventional cultures fail to identify the causative organism, molecular techniques, such as 16S rRNA gene analysis, could provide a definitive diagnosis, thereby enabling appropriate therapies.

## Introduction

Pelvic actinomycosis is a rare, chronic infection often associated with the prolonged use of intrauterine devices (IUDs) [1]. This condition is characterized by mass-forming, invasive behavior that can mimic malignant tumors, leading to diagnostic challenges [2-4]. *Actinomyces israelii*, an anaerobic filamentous bacterium, is one of the common causative pathogen, and the disease also occurs as a mixed infection with other anaerobes, where synergistic effects can exacerbate tissue invasion [5,6]. However, *Actinomyces *is difficult to detect using conventional culture techniques [7,8]. In such cases, molecular diagnostics such as 16S ribosomal RNA (rRNA) gene analysis provide a more sensitive approach, and when applied with next-generation sequencing (NGS), it enables simultaneous detection of multiple pathogens with higher sensitivity than conventional culture [9]. Herein, we present a case of severe pelvic actinomycosis and *Fusobacterium *infection that was difficult to differentiate from malignancy and featured hydronephrosis and intestinal infiltration due to extensive parametrial involvement following IUD removal. Furthermore, we demonstrate the utility of 16S rRNA gene analysis of formalin-fixed paraffin-embedded (FFPE) tissue in confirming the diagnosis of mixed infections caused by *Actinomyces *and *Fusobacterium*.

## Case presentation

**Figure 1:**
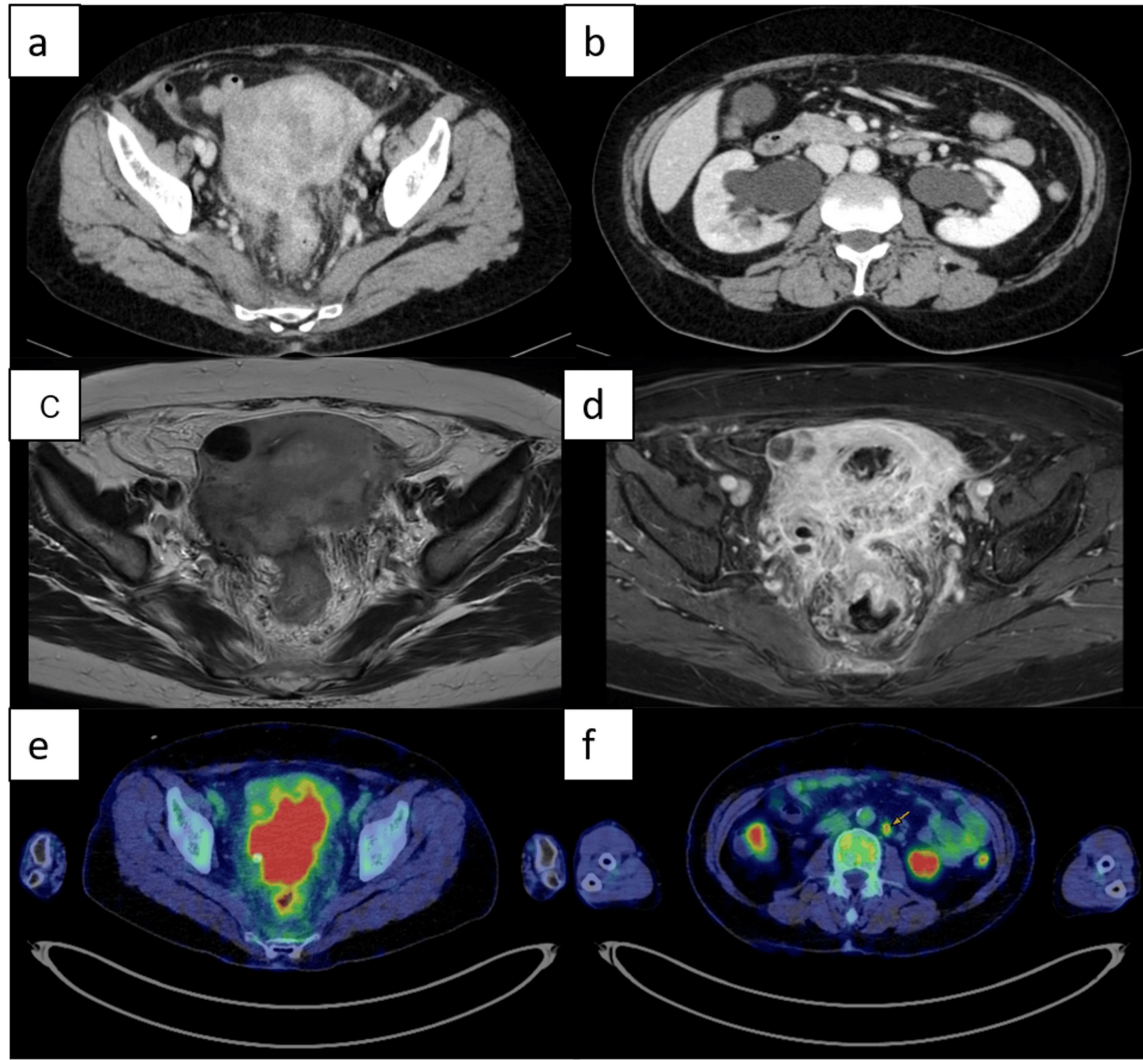
Imaging findings of the patient CT: computed tomography; PET-CT: positron emission tomography/computed tomography; MRI: magnetic resonance imaging; FDG: fluorodeoxyglucose (a, b) Contrast-enhanced CT showing an enlarged uterus and bilateral hydronephrosis and hydroureter. (c) T2-weighted MRI showing a heterogeneous signal intensity from the cervix to the body of the enlarged uterus. (d) Contrast-enhanced MRI showing heterogeneous contrast enhancement. (e, f) PET-CT showing intense FDG uptake in the mass extending from the cervix to the uterine body and in the paraaortic lymph node (yellow arrow)

A 65-year-old woman (gravida 3, para 3) presented to our emergency department with a chief complaint of lower abdominal pain and diarrhea. She did not have any other symptoms. The patient was 155 cm tall, weighed 61 kg, and had a BMI of 25.5 kg/m². She had experienced menopause at the age of 56 years, and had a medical history of hypertension, arrhythmia, and appendectomy for appendicitis. The patient had been previously diagnosed with uterine fibroids and was therefore undergoing follow-up care at a local clinic. Her medications included butylscopolamine bromide, bacterial butyric acid granules, pilsicainide hydrochloride hydrate, olmesartan medoxomil, and amlodipine besylate. Her family history included hypertension in her mother and breast cancer in her sister. The patient had undergone IUD insertion in 1984 following the birth of her third child, which had been removed eight months prior to presentation due to irregular bleeding and abdominal pain. After removal of the IUD, her symptoms resolved promptly. There were no clinical signs of infection during this period. On admission, her temperature was 36.7°C, blood pressure was 130/85 mmHg, and pulse was 81/minute. Pelvic examination revealed an enlarged uterus with poor mobility, whereas rectal examination detected a firm induration consistent with mass effect and adhesion. Laboratory findings indicated elevated inflammatory markers with a white blood cell count of 23,200/μL (normal: 3300-8600/μL) and C-reactive protein level of 4.89 mg/dL (normal: 0-0.14 mg/dL). Renal dysfunction was evident, with a blood urea nitrogen level of 23 mg/dL (normal: 8-20 mg/dL) and creatinine level of 1.30 mg/dL (normal: 0.46-0.79 mg/dL); however, tumor markers were all below cutoff values (carcinoembryonic antigen (CEA), 3.6 ng/ml; cancer antigen 19-9 (CA19-9), 11.2 U/ml; CA125, 19.2 U/ml; and squamous cell carcinoma (SCC), 0.8 ng/ml). The patient’s lactate dehydrogenase level was 166 U/L (normal: 124-222 U/L). Contrast-enhanced computed tomography (CT) revealed an enlarged uterus containing uterine fibroids with partial calcification, cervical enlargement, perirectal edema, and bilateral hydronephrosis (Figures 1a, 1b). Magnetic resonance imaging (MRI) revealed a mass lesion with heterogeneous signal intensity extending from the cervix to the body of the uterus, measuring 97 mm in maximum longitudinal diameter, with heterogeneous enhancement and regions suggestive of necrosis (Figures 1c, 1d). Positron emission tomography/CT demonstrated strong uptake in the uterine cervical-to-body mass and increased uptake in the para-aortic and bilateral iliac lymph nodes (Figures 1e, 1f).

**Figure 2:**
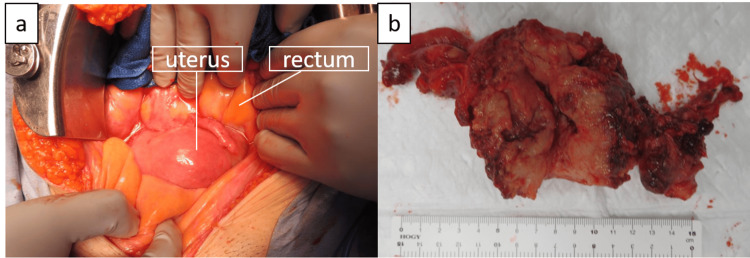
Operative findings Operative findings, including (a) firm adhesions around the enlarged uterus in the pelvic cavity and (b) resected specimen of the uterus

Cervical cytology revealed negative for intraepithelial lesion or malignancy (NILM). Barium enema examination subsequently revealed circumferential stenosis of the rectum without gross lesions, while total colonoscopy showed no mucosal invasion. Based on the results of these preoperative examinations, uterine sarcoma with extensive invasion was suspected, and open surgery was performed. Intraoperative findings included an enlarged uterus, extensive parametrial invasion, and firm rectal adhesions (Figure 2a). The right adnexa could not be identified as it was embedded in an invasive mass in the uterus; however, the left adnexa showed no abnormalities in either the fallopian tube or ovary. Type II radical hysterectomy, bilateral salpingo-oophorectomy, and pelvic and paraaortic lymph node biopsies were performed (Figure 2b).

**Figure 3:**
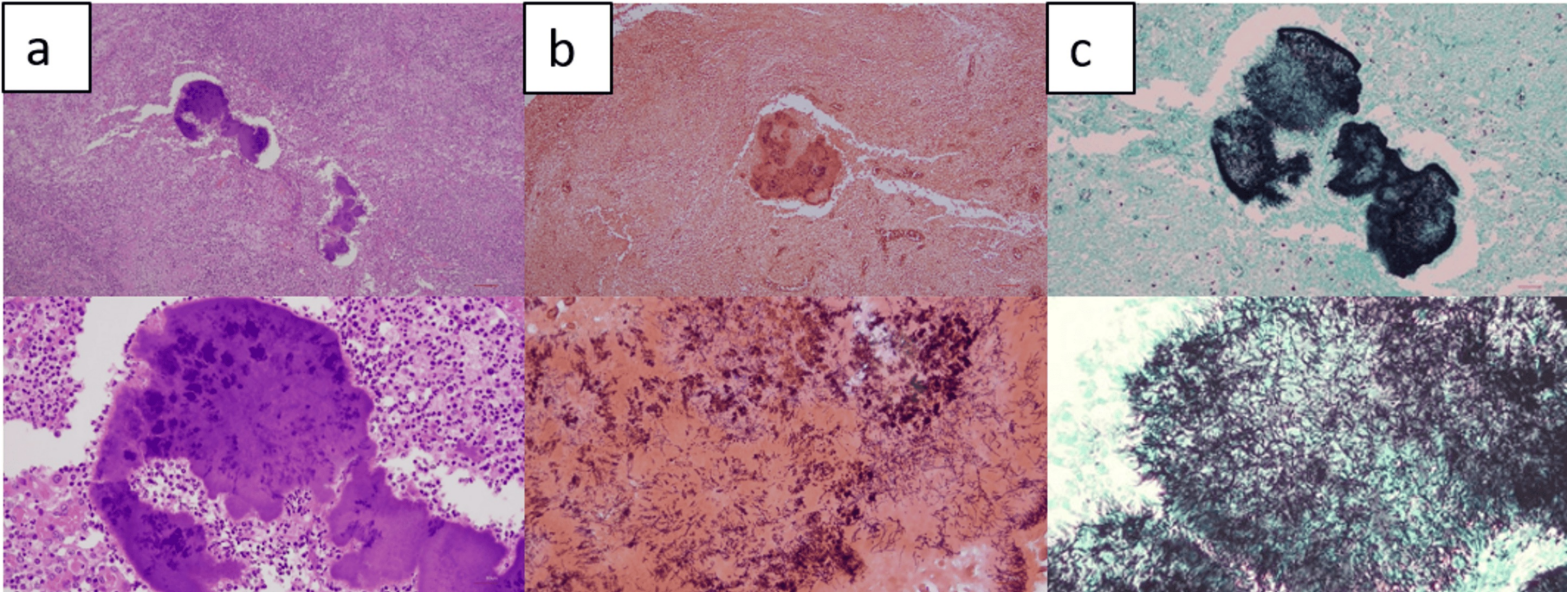
Histopathological findings Histopathological findings of the excised uterus. (a) Hematoxylin and eosin staining revealed bacterial colonies with extensive neutrophil infiltration. (b) Gram staining confirmed the presence of Gram-positive *bacilli*. (c) Grocott staining identified numerous black–brown filamentous bacterial colonies, presumed to be *Actinomyces*

However, rapid intraoperative pathological diagnosis using frozen section of the excised uterus indicated no malignant findings. The final pathological diagnosis further confirmed no apparent malignant tumor in the uterus, ovaries, and lymph node. However, hematoxylin and eosin staining of the excised uterus revealed bacterial colonies with extensive neutrophil infiltration and inflammatory fibrosis in the endometrium and myometrium (Figure 3a). Gram staining of the excised uterus confirmed the presence of Gram-positive *bacilli *(Figure 3b). Subsequent Grocott staining identified numerous black-brown filamentous bacterial colonies, presumed to be *Actinomyces* (Figure 3c).

The cultures of the ascitic fluid collected through the postoperative drain were monitored for three days, during which no growth was observed, confirming a negative result. NGS of 16S rRNA from FFPE tissue of the endometrium and myometrium was performed in three independent areas of the uterus. Genomic DNA was extracted from FFPE tissue sections using the NucleoSpin DNA FFPE XS kit (MACHEREY-NAGEL, Düren, Germany), and a negative control was processed in parallel without tissue. The V1-V2 region of the 16S rRNA gene was amplified by polymerase chain reaction (PCR), followed by purification using KAPA HyperPure Beads (Roche, Basel, Switzerland). Indexing was performed using the Nextera XT Index Kit v2 (Illumina, San Diego, CA, USA). Final libraries were sequenced on the Illumina MiSeq platform (Illumina, San Diego, CA, USA). NGS analysis revealed the presence of *Actinomyces israelii *and *Fusobacterium nucleatum *(Table 1). The patient received oral amoxicillin at a daily dose of 1,500 mg for seven weeks. Follow-up CT at six months demonstrated resolution of the hydronephrosis and no evidence of disease recurrence.

**Table 1:** 16S rRNA bacterial flora analysis rRNA: ribosomal RNA

Genus	Species	Occupancy rate
Area 1		
Fusobacterium	F. nucleatum	96.30%
Actinomyces	A. israelii	2.60%
Mesorhizobium	*Mesorhizobium *(unclassified)	0.30%
Cutibacterium	C. acnes	0.20%
Pseudomonas	P. donghuensis	0.20%
Chelatococcus	C. daeguensis	0.10%
Unclassified		0.20%
Others		0.10%
Area 2		
Fusobacterium	F. nucleatum	95.10%
Actinomyces	A. israelii	1.70%
Cutibacterium	C. acnes	0.70%
Pseudomonas	P. donghuensis	0.40%
Aquamicrobium	A. lusatiense	0.30%
Enterococcus	E. cecorum	0.30%
Paracoccus	P. marinus	0.20%
Others		0.20%
Area 3		
Fusobacterium	F. nucleatum	98.40%
Actinomyces	A. israelii	0.70%
Pseudomonas	P. donghuensis	0.40%
Cutibacterium	C. acnes	0.20%
Asticcacaulis	A. excentricus	0.10%
Enterobacteriaceae	*Enterobacteriaceae *(unclassified)	0.10%
Others		0.10%

## Discussion

The important clinical implications of this case include the following: First, mixed infection pelvic actinomycosis and Fusobacterium can develop even following IUD removal, causing hydronephrosis and intestinal infiltration through the extensive invasion of the parametrium, presenting a clinical picture similar to that of malignant tumors. Therefore, in patients with a history of IUD use, this disease should be considered, regardless of the patient’s current IUD status. Secondly, mixed infections, including synergistic relationships between *Actinomyces israelii *and *Fusobacterium nucleatum*, may play an important role in the pathogenesis of severe pelvic infection. The 16S rRNA analysis for FFPE tissue using NGS may aid in the diagnosis of pelvic severe combined infections.

*Actinomyces *is an anaerobic Gram-positive *bacillus* that normally inhabits the oral cavity and lower gastrointestinal tract [1]. *Actinomyces israelii *forms chronic suppurative granulomas [7]. In the field of gynecology, IUD placement has been reported as a risk factor of actinomycosis, which commonly develops due to long-term IUD retention [10]. Pelvic actinomycosis infection is generally diagnosed through postoperative histopathological examination in many cases [1]; however, in some cases, it can be diagnosed using cervical cytology, Gram staining of the abscesses, or culture [11,12]. Histological examination of Gram-positive filamentous organisms and sulfur granules strongly supports the diagnosis of actinomycosis [7]. However, this finding is not specific to actinomycosis [7]. A definitive diagnosis requires the direct isolation from clinical specimens, although success rates are low (>50% failure) due to prior antibiotic use, microbial overgrowth, or suboptimal anaerobic culture techniques [7,8]. A 16S rRNA gene analysis of FFPE tissue is highly valuable for the diagnosis of mixed infections. Indeed, there has been one prior case report in which mastoid infection with* Fusobacterium nucleatum* and *Actinomyces israelii *was not detected by culture of necrotic tissue but was detected by 16S rRNA gene analysis using NGS [5]. NGS of 16S rRNA gene analysis of FFPE tissues is crucial for the accurate diagnosis of culture-negative cases of suspected severe pelvic infection.

*Actinomyces *infections can be enhanced by combining infections with anaerobic bacteria. *Fusobacterium nucleatum* is an anaerobic, Gram-negative bacillus that commonly resides in the oral cavity and gastrointestinal tract. It is known to participate in mixed bacterial infections often acting synergistically with other pathogens [13-15]. In this case, a mixed infection of *Actinomyces israelii*, and *Fusobacterium nucleatum* was identified, indicating that infection establishment may have been facilitated more effectively than with *Actinomyces *infection alone. *Actinomyces *is a secondary colonizer of infections, potentially modifying the microenvironment of late-colonizing species [5,6]. This fact supports the hypothesis that the coaggregation of other bacterial species in actinomycosis prepares the microenvironment and promotes bacterial growth [11,12]. Supporting this hypothesis, that under in vitro conditions mimicking salivary biofilms, the growth of* Fusobacterium nucleatum *has been reported to require the presence of *Actinomyces* species [6]. Further, research has suggested that *Fusobacterium nucleatum* may form biofilms with *Actinomyces *species, potentially enhancing infection while evading host immune responses [6]. In the present case, it is thought that the mixed infection with *Actinomyces israelii* and *Fusobacterium nucleatum *caused advanced tissue invasion similar to that caused by malignant tumors. However, the potential synergistic effects of coinfection in this case remain speculative, as they are based on the obtained NGS results and previously reported studies. Further studies are needed to clarify this potential interaction.

Severe pelvic infection following IUD removal is difficult to differentiate from gynecological malignancies. Cases of pelvic actinomycosis developing despite IUD removal, or in the absence of IUD insertion, have previously been reported [2,16,17]. These cases were preoperatively diagnosed as malignant tumors, such as ovarian cancer [2-4]. Similarly, this case occurred following IUD removal and was preoperatively diagnosed as uterine sarcoma. The colonization of the female genital tract by *Actinomyces *spp. is greatly facilitated by IUD use [18]. Prior reports have indicated that IUD removal dramatically reduces genital colonization by *Actinomyces* and that the protective effect of IUD removal is proportional to the duration of patient use [19]. Although the exact mechanism is unknown, in some cases, *Actinomyces *colonization may persist even following IUD removal, potentially leading to severe pelvic infection. Therefore, when evaluating suspected cases of advanced malignancy, a history of IUD use should always be included in the patient interviews.

## Conclusions

This is an extremely rare case of severe mixed pelvic infection caused by *Actinomyces israelii *and *Fusobacterium nucleatum* after IUD removal. To our knowledge, this is the first reported case definitively diagnosed by 16S rRNA gene analysis of FFPE tissue using NGS, which might suggest that enhanced pathogenicity may be associated with a mixed infection of *Actinomyces israelii *and F*usobacterium nucleatum*. Obtaining a history of intrauterine device use is important for this disease, which commonly resembles malignant tumors on imaging. Furthermore, employing a combined approach of histopathological evaluation and 16S rRNA gene analysis with NGS enhances diagnostic accuracy and ensures a conclusive diagnosis.
